# Exploring landscape of drug abuse trends in Pakistan: A decade and a half of clinical laboratory big data analysis

**DOI:** 10.1371/journal.pgph.0004424

**Published:** 2025-05-05

**Authors:** Yousra Sarfaraz, Lena Jafri, Hafsa Majid, Nadeem Ullah Khan, Ayesha Sadiqa, Aysha Habib Khan, Imran Siddiqui, Sibtain Ahmed

**Affiliations:** 1 Medical College and Section of Chemical Pathology, Department of Pathology and Laboratory, Aga Khan University (AKU), Karachi, Pakistan; 2 Department of Emergency Medicine, Aga Khan University (AKU), Karachi, Pakistan; Centre of Biomedical Ethics and Culture, PAKISTAN

## Abstract

The utilization of urine testing for drugs of abuse has emerged as a valuable tool in discerning the evolving landscape of drug abuse patterns. The objective of this study was to determine the patterns of drug of abuse within the Pakistani population, incorporating demographic factors such as age, gender, and location. This observational study was conducted at the Department of Pathology and Laboratory Medicine, Aga Khan University from January 2008 to December 2022. A review of drugs of abuse screening data extracted from laboratory information system was carried out. The panel includes screening of common drugs, amphetamine (AMP), benzodiazepine (BZO), barbiturates (BAR), cannabinoids (THC), cocaine (COC), and opioids (OPI) as well as blood alcohol (ALC) levels. Mean, standard deviation, percentage and frequency were calculated by using STATA version 13.Over a period of 15 years, a total of 130, 859 tests were performed, with 89.5% being male and mean age of 32.4 ± 11.3 years, while seventy-seven percent of the specimens (n = 101, 648) came from the province of Sindh. Of the total 6270 (4.8%) were screened positive for at least one drug, THC (n = 3626, 57.8%) was the most frequently positive test, followed by OPI (n = 1000, 16%), ALC (n = 671, 10.7%), COC (n = 31, 0.5%), AMP (n = 45, 0.7%), BAR (n = 35, 0.6%) and BZO (n = 862, 13.7%). Yearly trend analysis shows an increasing number of THC tests requested over time, with THC exhibiting the highest positivity rate, followed by BZO, OPI, and ALC.The 15-year patterns depict the rising prevalence of drug consumption which is subsequently increasing the demand for drug screening tests. The study highlights Pakistan’s growing drug prevalence and calls for targeted policies to address its use, including strengthened prevention programs, improved regulation and better management and rehabilitation access.

## Background

Drug abuse is a growing concern, with a variety of narcotics causing mild to extreme calamities, particularly in South Asia. This region of the world is evidently reported to be more exposed to opium and cannabis (marijuana) [1]. Stimulants, sedatives, hallucinogens, and opioids are among the substances that are abused worldwide, including in Pakistan. While highly addictive, narcotics, such as benzodiazepines (BZO) or opioids, are clinically significant analgesics and sedatives; however, the abuse of these drugs frequently leads to the development of tolerance, dependence, and overdose [2]. According to the ‘2004 Abused Drug Epidemic’, cannabis remained the most commonly abused substance in Southeast and Southwest Asia, with opium and heroin preferred due to accessibility and low price [3]. Also, cannabis, BZO, opiates (OPI) and barbiturates (BAR) hold significant medical effects, but their constant recreational use has overshadowed their potential benefits [4,5,6].

The Control of Narcotic Substances Act, 1997 is Pakistan’s legislation aimed at controlling and regulating narcotic drugs and psychotropic substances. The Anti-Narcotics Force enforces drug laws and participates in drug abuse prevention and treatment initiatives. The National Anti-Drug Abuse Policy emphasizes awareness, demand reduction, and treatment facilities. Public health initiatives, including screening in healthcare facilities, industries, educational institutions, and NGOs, also support national efforts against drug abuse. Despite the existence of legislation aiming at regulating drug misuse in Pakistan, their implementation confronts various problems, resulting in inadequate and inconsistent effects. Alcohol, while not a narcotic, is included in this current study because of its significant psychoactive effects and public health concerns. In Pakistan, where alcohol drinking is outlawed, identifying its use and impact is critical for conducting a thorough analysis of substance use. Retrospective analysis of alcohol testing will help identify trends in alcohol consumption, evaluate control policies, inform policymakers, and identify high-risk groups for targeted interventions, enabling informed decisions on future regulations and public health challenges.

In Pakistan, commonly consumed drugs of abuse include alcohol (ALC), amphetamine (AMP), BZO, marijuana, cocaine (COC), and OPI [7]. These are locally available as ICE, meth, K, blues, hashish, weed, and Ganja. Pakistan faces significant narcotics industry challenges, with 3.5 million drug abusers reported in 2005, which is increasing by 7% annually, according to the World Drug Report 2000 and 2005 United Nations Drug Control Programme Project survey [8]. Around 71.5% of these drug addicts are aged 35 years or older, with the highest proportion aged between 20–30 year [9].

Pakistan lacks a comprehensive national database for monitoring substance abuse trends, relying on survey-based investigations. Establishing a robust database is crucial for developing precise prevention and intervention approaches. With a deeper comprehension of prevalent drug patterns, more targeted and efficient interventions can be developed to combat substance abuse. The objective of the current study was to evaluate the distribution patterns of positive drug abuse cases detected in urine samples and ALC detected in whole blood as analyzed by the clinical laboratory of Aga Khan University (AKU), Pakistan.

## Methodology

This observational study was conducted in the Section of Chemical Pathology, Department of Pathology and Laboratory Medicine, AKU. Presently the clinical laboratory network of AKU encompasses laboratories within AKU tertiary and secondary care campuses, along with more than three hundred collection points/ phlebotomy centres in more than ninety districts across Pakistan, making it one of the largest of its kind in the country. This extensive network of laboratories and phlebotomy centers caters not only to individuals within the AKU health system but also to patients and physicians across Pakistan. Pakistan, as a whole, boasts a diverse population of over 220 million people, hailing from various ethnic backgrounds, and is dispersed across four provinces [Balochistan, Khyber Pakhtunkhwa (KPK), Punjab, Sindh] and Azad Jammu and Kashmir (AJK). The data concerning drugs of abuse were extracted from the Laboratory Information System (LIS) of the Section of chemical Pathology, Department of Pathology and Laboratory Medicine, AKU. Drugs of abuse detected in urine samples included AMP, BZO, BAR, cannabinoids (THC), COC, and opioids (OPI) as well as blood ALC levels.

Information regarding the presence of drugs of abuse in urine/blood samples was accessed on 12/07/2023 from the LIS of AKU. The data collection spanned from January 2008 to December 2022. The laboratory data encompassed an analysis of individual drugs of abuse, the comprehensive panel of drugs of abuse and quality control (QC) data of all the drugs of abuse studied. We aggregated the data at the individual level that each person is represented by a single record. In circumstances where many entries existed owing to data input errors or other abnormalities, we used deduplication techniques to save only the most accurate and first record for each individual. Cases with missing or incomplete information were excluded.

Analysis of urine drugs of abuse and blood ALC levels were performed on Dimension EXL, using enzyme multiplied immunoassay technique (EMIT) methodology. Internal QC material was run with every batch of patient samples analyzed. The laboratories at AKU have implemented a rigorous QC regimen that adheres to the guidelines set forth by the Clinical Laboratory Standard Institute (CLSI). Furthermore, these laboratories have obtained accreditation from esteemed organizations such as the College of American Pathologists (CAP) and the Joint Commission International. The implementation of these rigorous protocols has led to the standardization of the data produced within this system. This standardization facilitates effective consolidation, analysis, and comparison of data. Proficiency testing surveys from CAP for drugs of abuse in urine and blood ALC were conducted from 2014 onwards, while prior to that, Bio-Rad’s External Quality Assurance Services (EQAS) surveys were analyzed.

The data was analyzed by Stata version 17. Descriptive statistics are given as the mean and standard deviation. Frequencies and percentages are generated for qualitative data. The age ranges monitored for the test being ordered were classified into adolescence (<18), early adulthood (18–38), middle age (39–59), and old (>=60). The association between positivity rates of DOA for different age groups and genders were assessed by Chi Square test. The Global Administrative Areas Database (GADM) (www.gadm.org) was used to find Pakistan-specific spatial coordinates and divisions. To establish spatial coordinates for visiting locations within Sindh, Punjab, Balochistan, KPK and AJK where the specimens (urine and blood) were given for analysis, the laboratory and hospital database was utilized. For cases with missing details and verification needs, Google Maps was employed to obtain accurate spatial information. Based on the map supplied by Global Administrative Areas (GADM), frequencies and individual counts were grouped according to the spatial subdivision of provinces. Frequencies and percentages of positive results were used to produce a provincial-level choropleth map using Environmental Systems Research Institute Geographic Information System (ArcGIS) software: https://server.arcgisonline.com/ArcGIS/rest/services/World_Terrain_Base/MapServer.

## Results

### Baseline characteristics and summary of specimens analyzed

Over a fifteen-year timeframe, a total of 130, 859 tests were screened for seven different types of drugs of abuse. The overall mean age of the screened population was 32.4 ± 11.3 years, with males 117,099 (89.5%) being tested more frequently than females 13,760 (10.5%). The majority of specimens were received from the province of Sindh (77%; n = 101, 648). The distribution of drugs of abuse tests received from all provinces is shown in Table 1. The most frequently requested drug test was THC 35.9% (n = 46,924), followed by OPI 30.9% (n = 40,394), ALC 14.4% (n = 18,856), COC 6.7% (n = 8,802), AMP 5.6% (n = 7,377), BAR 4.2% (n = 5,531) and BZO 2.3% (n = 2,975). The proficiency testing survey record of urine toxicology and blood ALC test shows 95% - 100% of results accuracy, validating the proficiency of our testing methodology, reflecting reliability and precision of our documented results.

**Table 1:** Total number of tests ordered, and positive cases reported categorized on the basis of gender and geographical distribution.

Variables		Total ordered tests	Positive for Drugs of Abuse n (%)							
		n (%)	Overall	ALC	AMP	BAR	BZO	THC	COC	OPI
**Overall**		130, 859 (100)	6270 (4.8)	671 (10.7)	45 (0.7)	35 (0.6)	862 (13.7)	3626 (57.8)	31 (0.5)	1000 (16.0)
**Gender**	**Males**	117,099	5874 (5.0)	640 (10.9)	43 (0.7)	24 (0.4)	602 (10.3)	3580 (61.0)	30 (0.5)	954 (16.2)
	**Females**	13,760	397 (2.3)	31 (7.8)	2 (0.5)	11 (2.8)	260 (65.5)	46 (11.6)	1 (0.3)	46 (11.6)
**Geographical Distribution**	**AJK**	51	9	1 (11.1)	–	–	–	7 (77.8)	–	1 (11.1)
	**Balochistan**	914	75	–	2 (2.7)	–	2 (2.7)	54 (72.0)	–	17 (22.7)
	**KPK**	5462	237	5 (2.1)	2 (0.8)	–	13 (5.5)	172 (72.6)	–	45 (10.0)
	**Punjab**	22,784	1542	66 (4.3)	15 (1.0)	2 (0.13)	85 (5.5)	1049 (68.1)	8 (0.5)	317 (20.5)
	**Sindh**	101,648	4408	599 (13.6)	26 (0.6)	33 (0.7)	762 (17.3)	2344 (53.2)	23 (0.5)	621 (14.1)

A Chi-square test was conducted to examine the association between the drug categories (ALC, AMP, BAR, BZO, THC, COC and OPI) and the total outcome indicates a significant association between the drug categories and the total outcome (p < 0.001). To assess the relationship between gender (female and male) and the outcomes a chi-square test was performed. The test revealed a statistically significant association (p < 0.001),.A significant association in chi-square test (p<0.001) was also obtained for the geographical location based result.

### Drugs of abuse screen positive trend over the years

**Fig 1:**
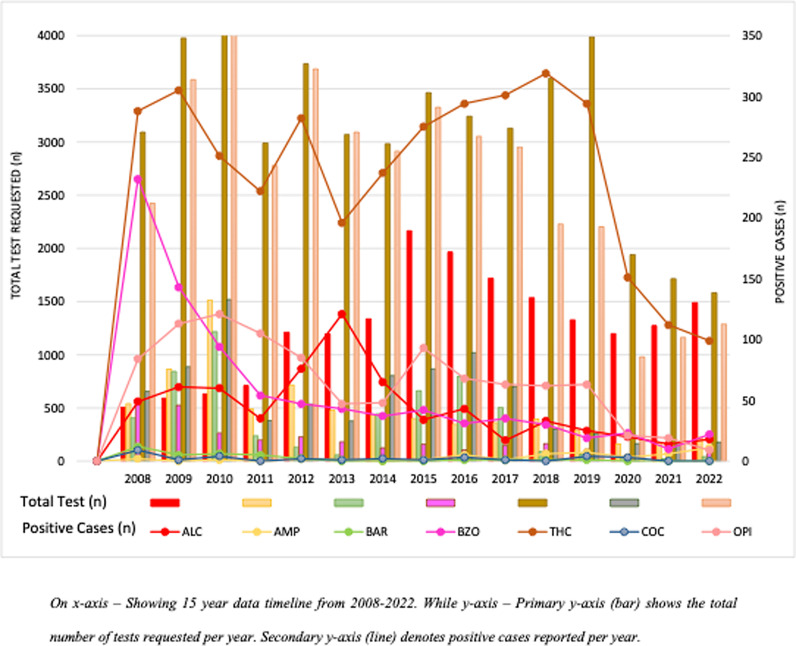
Yearly data of drugs of abuse screen positive against total volume from 2008-2022; from Clinical Laboratory of Aga Khan University Pakistan.

The percentage for total screen positives reported was 4.79% (n = 6271) with THC (57.8%) being the most frequently positive, followed by OPI (15.9%) and BZO (13.7%). A Chi-square test was conducted to examine the association between the drug categories (ALC, AMP, BAR, BZO, THC, COC and OPI) and the total outcome. The results indicated a significant association between the drug categories and the total outcome (p < 0.001). The gender and geographical distribution of positive screen tests is shown in Table 1. On yearly trend analysis, the number of THC tests requested has increased over the years, with the highest positivity rate, followed by BZO, OPI, and ALC. Fig 1 shows the volume of testing, and positive screen test for each drug of abuse over a fifteen-year period (S1 Table).

### Positivity according to age and gender

In gender-based patterns, male consumption of drugs and the number of tests ordered were comparatively higher, as shown in Table 1. On gender comparison females were more frequently screened positive for BZO (*p* value <0.001), while THC were more frequently detected in males (*p* value <0.001). Table 2 displays data on screen positive drugs of abuse test results across various age groups and genders. The age group 18–38 years had the highest rate of positivity, followed by the age group 39–59. In the less than 18-year-old age group, the majority of specimens screened positive (90.4%) were from males.

**Table 2:** Drugs of abuse percentage of positive results categorized by age and gender.

Age	Total Screen Positive	Female (%)	Male (%)
n = 6,270	n = 397	n = 5873
<18	385 (6.1%)	37 (9.6%)	348 (90.4%)
18-38	4,441 (70.8%)	214 (4.8%)	4,227 (95.2%)
39-59	1,239 (19.8%)	88 (7.1%)	1,151 (92.9%)
>=60	205 (3.3%)	58 (28.3%)	147 (71.7%)

Chi square test significant association (p<0.001) is used, in drug positive female, are significantly associated with age categories. As well as male also show significant difference between age categories with positive drug screening result.

### Province wise distribution of drugs of abuse

**Fig 2:**
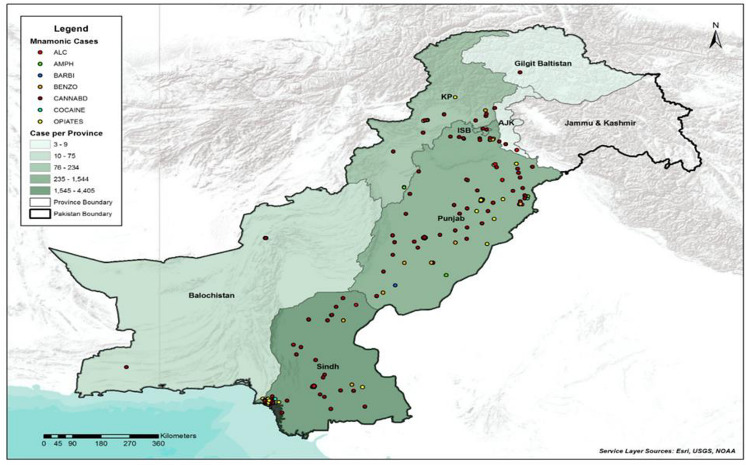
Chloropeth map showing the density of drugs of abuse testing and common positive screen drugs in each province of Pakistan. **Legend:** The link to the base layer of the map is: shows the volume of testing, and positive screen test for each drug of abuse over a fifteen-year period https://server.arcgisonline.com/ArcGIS/rest/services/World_Terrain_Base/MapServer. Additionally, the link to the shapefile is https://data.humdata.org/dataset/cod-ab-pak.

The geographical distribution for positive screen and density of drugs of abuse testing is shown in Fig 2. Sindh provided the most samples, followed by Punjab, KPK, Balochistan and AJK. Sindh recorded the highest number of positive cases as per provincial data analysis (table 1). Notably, BZO stood out as the most frequently detected drug, accounting for 34.2% of positive results. Following BZO, THC exhibited a relatively high positivity rate at 6.7%, while ALC showed a positive rate of 5.5%. KPK and Punjab reported higher numbers of positive cases after Sindh, with 237 and 1542 positives reported from 2008 to 2022, respectively. Even though the number of reported cases within AJK was low, the positivity rate was still around 35% higher in comparison with other provinces.

## Discussion

World drug report 2014, reported that 162–324 million (3.5% to 7%) of the world’s population aged 15–64, used illicit drugs like cannabis, OPI, COC, or amphetamine-type stimulants [10]**.** While recent reports of 2023 state that the number of drug abusers increased, from 240 million in 2011–296 million in 2021, accounting for 5.8% of the global population aged 15–64 [11].

The ongoing study incorporates valuable insights, including data from a 2008 investigation that conducted 17,714 urine toxicology tests where BZO emerged as the most prevalent drug among the subjects [12]. The hike in drug abuse faced by Pakistan is significant being a country where usage of these drugs such as cocaine, cannabinoids are illegal. The overall positivity rate (4.8%) from our data were comparable to US positivity rates for drugs of abuse, which was 4.6% in 2021 according to the Quest Diagnostics Drug Testing Index [13].

In this decade-and-a-half-long study of clinical laboratory data, it is evident that THC was the most frequently ordered test among the urine toxicology test panel, with a positivity rate of over 50%. In Pakistan, research suggests that around 6.7 million individuals are engaged in drug abuse, with 4.25 million among them experiencing dependency and requiring extended residential treatment. Despite these alarming figures, there is a lack of preventive measures or policies addressing drug offenses. Among the drug abusers surveyed, 78% attributed their involvement to factors like peer pressure, family conflicts, stress, and financial troubles. Additionally, 24% of the participants acknowledged turning to substance abuse out of curiosity and a pursuit of pleasure [14].

Previous studies have reported that mostly males are more inclined toward substance abuse in comparison with females [15,16,17,18]. Our study also forges the same hypothesis as the number of males was dominant in our data, the consumption was common in middle-aged men (70%).

Pakistan is considered to be one of the largest producers of THC. With an estimated 219 million users (4.3% of the adult population worldwide), cannabis will continue to be the most commonly used substance in 2021. Though men make up the majority of cannabis users worldwide (about 70%), the gender gap is narrowing in some subregions, where women make up 42% of cannabis users [11]. Within our study setup, THC is the most frequently reported drug, with distribution patterns varying among provinces. Cannabinoids emerged as the most frequently tested drug within AJK, indicating a notable prevalence, while other drugs were either not reported or reported at minimal levels within this region. The positivity rate, although varying in Sindh and Punjab, consistently showed THC as the most frequently ordered test, suggesting a higher positivity. In terms of gender-based results, reports of THC consumption remained prominent among males aged over fifteen years, while females were also reported with a lesser margin of difference.

As suggested by our laboratory data, BZO is the second most highly consumed drug, in many parts of the world there has been an increase in the use of psychoactive substances, primarily BZO and BAR. When BZO were introduced into clinical practice (in the 1950s), they were considered efficacious minor tranquilizers, largely devoid of unwanted side effects, in contrast to the barbiturates. Continued use of BZO has often been oversimplified, and they still continue to be the most widely prescribed psychoactive drugs worldwide. Popularity of BZO among drug addicts has grown as they are not only inexpensive, but also treat a variety of conditions such as sleep disturbances and anxieties. They have been used with some frequency in drug abusers, mostly in the context of poly drug abuse. Due to the possible behavioral and physical side effects, as well as the negative impacts on the economy and society, psychoactive medicine use requires healthcare practitioners to prescribe prescriptions carefully. In Pakistan, most of the medications, even those that are psychotropic, are widely accessible without a doctor’s prescription that may result in significant health and societal risks. Pakistan is reported to have a 41% drug misuse rate [19] for BZO and another study reported that around 84% were cases of self-poisoning [20]. Women are more prone to exhibit depressive and anxiogenic behavior, the data is depicting the same. Benzodiazepine abuse is found more commonly within females in comparison with male with almost twice a difference in positivity rate 44.5% and 25.2% and several previous studies have comparatively reported same that men were less prone than women to abuse benzodiazepines [21,22,23].

ALC misuse accounts for 3.5% of global drug abuse and is the fourth leading cause of disability in underdeveloped nations [24]. It is the third most common substance abuse tested globally, with 5.8% of the population testing positive. According to the WHO survey from Pakistan gender differences vary by 17% to 1% [25]. ALC is consumed intentionally as a “drink of choice” at social events [26]. The study conducted in Karachi estimates that 28% stress coping, 47% social influence, and 45% marital or parental problems are the factors which contribute to initiation of ALC use [27,28]. The current study shows ALC is the third most requested test and fourth most frequently reported positive substance abuse in Pakistan, with male consumption consistently insignificantly higher than female consumption.

In 2021, an estimated 60 million people, primarily heroin users, engaged in non-medical opioid use, with 31.5 million using OPI. Pakistan’s 2006 National Assessment Report on Problem Drug Use reveals 628,000 estimated opiate users, with nearly 77% using heroin **[**29]. A study conducted in 2019 reported that around 60.7% of the study population abused heroin, marking nearly 80% of the study cohort from Peshawar [14]. Similarly, OPI misuse was readily reported within AJK in our study. From the current study it is evident that OPI is the third most ordered test within this region. However, Punjab excels in the positivity rate with nearly treble of positive sample in contrast with Sindh. The problem is aggravated by heroin (opiate) use as it is cheap and readily available due to the country’s widespread border engagement with Afghanistan, and so consumption is hiking as the day passes.

Stimulant drugs COC and AMP are consumed by 0.4% and 0.6% of the global population [12,15]. Our findings show that no rising trend of COC and AMP positivity was seen over the years. These findings concur with previously reported findings [12]. Amphetamine consumption was reported to be equal among females and males in the current study, and there has been a decline in the number of tests ordered over the years. The current study highlights Pakistan’s increasing drug prevalence and calls for targeted policies to address cannabis use. It suggests strengthened prevention programs, improved regulation, better access to treatment, regional disparities, and youth-centric interventions. The study emphasizes the need for education, community organizations, and social services to promote awareness and support.

## Limitations

This study has a few limitations. The biochemical analyses were merely for screening purposes, and no confirmatory assays were performed. The absence of confirmation testing raises the possibility of false positives or negatives, which could lead to incorrect conclusions concerning the presence or absence of substances of abuse. It’s a single center study not representative of the entire population. However, AKU has diverse laboratory setup, that spans across nearly all provinces. As a retrospective study, we did not specifically address the potential biases introduced by the overrepresentation of certain geographic locations or gender groups. The information about exposure (drug users) and non-exposure (non-users) we cannot calculate odds ratio. This could impact the generalizability of the findings, particularly with respect to regional or gender-specific trends and future studies must be conducted with more balanced sampling strategies to help mitigate such biases.

Cases with missing or incomplete data were excluded from the analysis as it constituted a small proportion of the dataset and no formal statistical tests were conducted, the minimal extent of missing data suggest limited impact on the findings. However, results are interpreted with caution, and future studies is considered for formal analysis of missing data.

## Conclusion

The decade-and-a-half data from the clinical laboratory illustrates a pattern indicating an increase in both drug positivity distribution and testing trends over these years. However, the affected age group and gender have remained consistent. Adults, especially middle-aged men, dominate these groups, suggesting a correlation with sociodemographic, economic, and emotional instacocbility as potential factors contributing to drug consumption issues. In a developing country like Pakistan, these issues impact both mental and financial health, exacerbated by the availability of drugs at lower prices, making national-level consumption more vulnerable [30]. While this data provides insights into drug usage and testing awareness trends within the population, the need for a national database to explore patterns of drug abuse remains an imperative obligation. Furthermore, based on these findings several community programs, effective treatments option and gender specific support programs can be designed, the trend can provide critical insight to the key stakeholders, including healthcare providers, policymakers, and NGOs, in developing targeted and effective strategies to address drug of abuse growing issue within in Pakistan. These insights emphasize the need for evidence-based decision-making, prioritizing interventions that are both contextually relevant and resource-efficient.

## Supporting information

S1 Table Drug testing volume and positivity rates in clinical laboratory samples at Aga Khan University (from 2008-2022). (XLSX)
